# Improving Staff Knowledge of Alcohol Withdrawal, Assessment and Treatment Pathway for Patients Detained in Place of Safety

**DOI:** 10.1192/bjo.2026.11270

**Published:** 2026-06-30

**Authors:** Sunday Adeoye, Odira Anakebe, Sarah Bradbury

**Affiliations:** Humber Teaching NHS Foundation Trust, Hull, United Kingdom

## Abstract

**Aims:**

Alcohol withdrawal can potentially result in a life-threatening condition. This requires early recognition, prompt assessment and treatment. The National and Humber Trust guidelines emphasise use of validated tools such as Severity of Alcohol Dependence Questionnaire (SADQ) and Clinical Institute Withdrawal Assessment for Alcohol–Revised (CIWA–Ar) to guide treatment decisions.

An incident of alcohol withdrawal in the 136 suites, in which the patient’s symptoms were not recognised early and treatment was inadequate, alongside the presence of new staff within the team and informal discussions with staff, highlighted variability in staff knowledge regarding assessment tools and the treatment pathway.

The aim of the project was to improve staff confidence and knowledge in assessing and managing alcohol withdrawal in line with Trust Guidelines as measured by the pre-and post-intervention questionnaire.

**Methods:**

The project followed the PDSA cycle of Quality Improvement:

Plan: Improve staff knowledge and confidence in managing alcohol withdrawal.

Do: We conducted a teaching session with staff on alcohol withdrawal symptoms, assessment tools and how they are used and required treatment.

We also created a flowchart to summarise the diagnosis and treatment of alcohol withdrawal. These flowcharts were pasted in suite 136 for staff to use as quick reference.

Study: Post-intervention results showed improved staff knowledge and confidence. Flowcharts were frequently used as quick reference points, and lectures enhanced understanding of assessment and management.

Act: Continue teaching and display of flowcharts. Future cycles may focus on evaluating clinical outcomes of alcohol withdrawal management.

**Results:**

The results showed that the intervention carried out led to significant improvement in staff knowledge of alcohol withdrawal assessment tools, treatment pathways, and guideline access.

The project showed that combining lectures and visible flowcharts effectively improved staff knowledge and confidence in managing alcohol withdrawal. Ongoing reinforcement and feedback are essential to ensure that improved knowledge translates to consistent practice.

**Conclusion:**

This project helps staff to manage alcohol withdrawal more efficiently thereby saving time. It also helps to optimise use of resources. Early recognition and treatment initiation helps to prevent complication thereby cost saving.

